# Dissolved hyperpolarized xenon‐129 MRI in human kidneys

**DOI:** 10.1002/mrm.27923

**Published:** 2019-08-09

**Authors:** Jorge Chacon‐Caldera, Adam Maunder, Madhwesha Rao, Graham Norquay, Oliver I. Rodgers, Matthew Clemence, Claudio Puddu, Lothar R. Schad, Jim M. Wild

**Affiliations:** ^1^ Computer Assisted Clinical Medicine, Medical Faculty Mannheim Heidelberg University Mannheim Germany; ^2^ POLARIS, Unit of Academic Radiology University of Sheffield Sheffield United Kingdom; ^3^ Philips Healthcare Guildford United Kingdom

**Keywords:** ^129^Xe, hyperpolarized xenon, kidney MRI, physiology, renal

## Abstract

**Purpose:**

To assess the feasibility of using dissolved hyperpolarized xenon‐129 (^129^Xe) MRI to study renal physiology in humans at 3 T.

**Methods:**

Using a flexible transceiver RF coil, dynamic and spatially resolved ^129^Xe spectroscopy was performed in the abdomen after inhalation of hyperpolarized ^129^Xe gas with 3 healthy male volunteers. A transmit‐only receive‐only RF coil array was purpose‐built to focus RF excitation and enhance sensitivity for dynamic imaging of ^129^Xe uptake in the kidneys using spoiled gradient echo and balanced steady‐state sequences.

**Results:**

Using spatially resolved spectroscopy, different magnitudes of signal from ^129^Xe dissolved in red blood cells and tissue/plasma could be identified in the kidneys and the aorta. The spectra from both kidneys showed peaks with similar amplitudes and chemical shift values. Imaging with the purpose‐built coil array was shown to provide more than a 3‐fold higher SNR in the kidneys when compared with surrounding tissues, while further physiological information from the dissolved ^129^Xe in the lungs and in transit to the kidneys was provided with the transceiver coil. The signal of dissolved hyperpolarized ^129^Xe could be imaged with both tested sequences for about 40 seconds after inhalation.

**Conclusion:**

The uptake of ^129^Xe dissolved in the human kidneys was measured with spectroscopic and imaging experiments, demonstrating the potential of hyperpolarized ^129^Xe MR as a novel, noninvasive technique to image human kidney tissue perfusion.

## 1 INTRODUCTION

The early diagnosis and characterization of kidney diseases is a nontrivial task due to hyperfiltration, a compensatory mechanism in which the overall filtration rate is initially maintained despite a decreased function of 1 of the kidneys.^1^, ^2^ Conventional methods to assess kidney function rely on the quantification of glomerular filtration rates that reflect the filtration efficiency of both kidneys, overlooking compensation mechanisms. These exams involve the administration of a marker whose concentration is monitored in plasma or urine for several hours to evaluate the clearance achieved by the kidneys. Tracers can be chelated with radioisotopes to allow detection by imaging. However, imaging‐based methods have been proven to be less accurate than clearance methods with the additional disadvantage of the ionizing radiation dose.^3^ Several MR‐based methods have been used to study renal physiology with the purpose of finding biomarkers that reflect early changes in kidney diseases. Notable examples include dynamic contrast‐enhanced MRI (DCE‐MRI)^4^, ^5^ and arterial spin labeling (ASL).^6^, ^7^

DCE‐MRI allows for the measurement of perfusion and filtration, both of which are key parameters in the assessment of renal function. DCE‐MRI, however, is an invasive technique that requires an injection of gadolinium as a contrast agent. Historically, some cases of nephrogenic system fibrosis in kidney patients have been reported after the use of gadolinium,^8^ and it is contraindicated for renal studies in several institutions. Moreover, new evidence suggests that gadolinium is deposited in the brain.^9^

ASL is a noninvasive alternative to study renal physiology where the blood entering the kidneys is used as a contrast agent. Recently, it has been reported that the perfusion in the cortex measured with ASL is affected by kidney diseases.^10^ This promising technique is safe and noninvasive but it has not been established in the clinical routine and there is considerable variability among perfusion values reported in the literature.^11^, ^12^ There are several challenges that are involved in ASL in the kidneys. For instance, the method relies on image subtraction (control and label), which affects the contrast‐to‐noise ratio of the output data. This is normally alleviated by averaging multiple images that require adequate motion correction and registration of the input images to account for breathing motion and pulsation. Additionally, apart from the sequence and imaging parameters, there are a multitude of parameters that need to be accurately adjusted, such as position and characteristics of the labeling plane (delay, duration, B_1_, and thickness), T_1_ of blood, and background suppression, to name the most relevant.^13^

The search for novel methods and biomarkers to study renal physiology is currently ongoing research in MRI. For example using novel water and ^13^C,^15^N_2_‐urea hyperpolarization methods for renal angiography and perfusion was recently demonstrated in pigs’ kidneys in vivo after the delivery of the contrast agent was injected directly in the artery.^14^, ^15^

Hyperpolarized ^129^Xe MRI is an emerging technique to obtain high contrast‐to‐noise ratio spectroscopic and imaging information. Xenon‐129 is an inert gas that can be safely inhaled and has been used as a contrast agent primarily for functional lung imaging, to date.^16^ However, recent works have demonstrated that inhaled hyperpolarized ^129^Xe dissolves in the blood in the lungs and can then be imaged in distant organs such as the brain in humans.^17^, ^18^ The kidney is one of the most well perfused organs in the body, receiving about 20% of the cardiac output through the descending aorta. With a T_1_ in blood of approximately 8 seconds^19^ and a lung–kidney circulatory transit time of a few seconds, the dissolved ^129^Xe can reach the kidneys sufficiently polarized for in vivo MR detection, as demonstrated previously in preliminary work at 1.5 T with MRI^20^ and dynamic spectroscopy.^21^

In this work, we demonstrate MR spectroscopy and imaging of dissolved hyperpolarized ^129^Xe in kidneys of healthy volunteers at 3 T, with a view to enable clinical studies related to renal physiology. We performed dynamic and spatially resolved spectroscopy in the abdomen. Based on the findings from dynamic spectroscopy of ^129^Xe uptake in the kidneys, dynamic imaging was then performed with 2 RF coil setups: a single quadrature transceiver coil and a transmit‐only receive‐only (TORO) coil array.^22^ Spoiled gradient echo (SPGR) and balanced steady state free precession (bSSFP) sequences were both evaluated for renal ^129^Xe imaging.

## 2 METHODS

Hyperpolarization of 80% enriched ^129^Xe gas was performed in house using the spin‐exchange optical pumping method as previously described^23^ under the corresponding author's UK MHRA manufacturing regulatory license. For imaging and local spectroscopy, 1‐L Xe doses were prepared while 0.5 L of Xe topped to 1 L with medical‐grade N_2_ doses were used for the dynamic spectroscopy. All in vivo experiments were performed with the approval of the UK national research ethics committee. Three healthy male volunteers (ages 29, 32, and 35 years old) participated in this study with prior written consent. The volunteers were informed to inhale the hyperpolarized ^129^Xe gas and hold their breath for as long as could be comfortably tolerated (maximum of approximately 40 seconds) with continuous monitoring of heart rate and finger pulse oxygenation. All MR measurements were performed on a 3T system (Ingenia; Philips Healthcare, Amsterdam, Netherlands).

### 2.1 Radiofrequency coils

**Figure 1:**
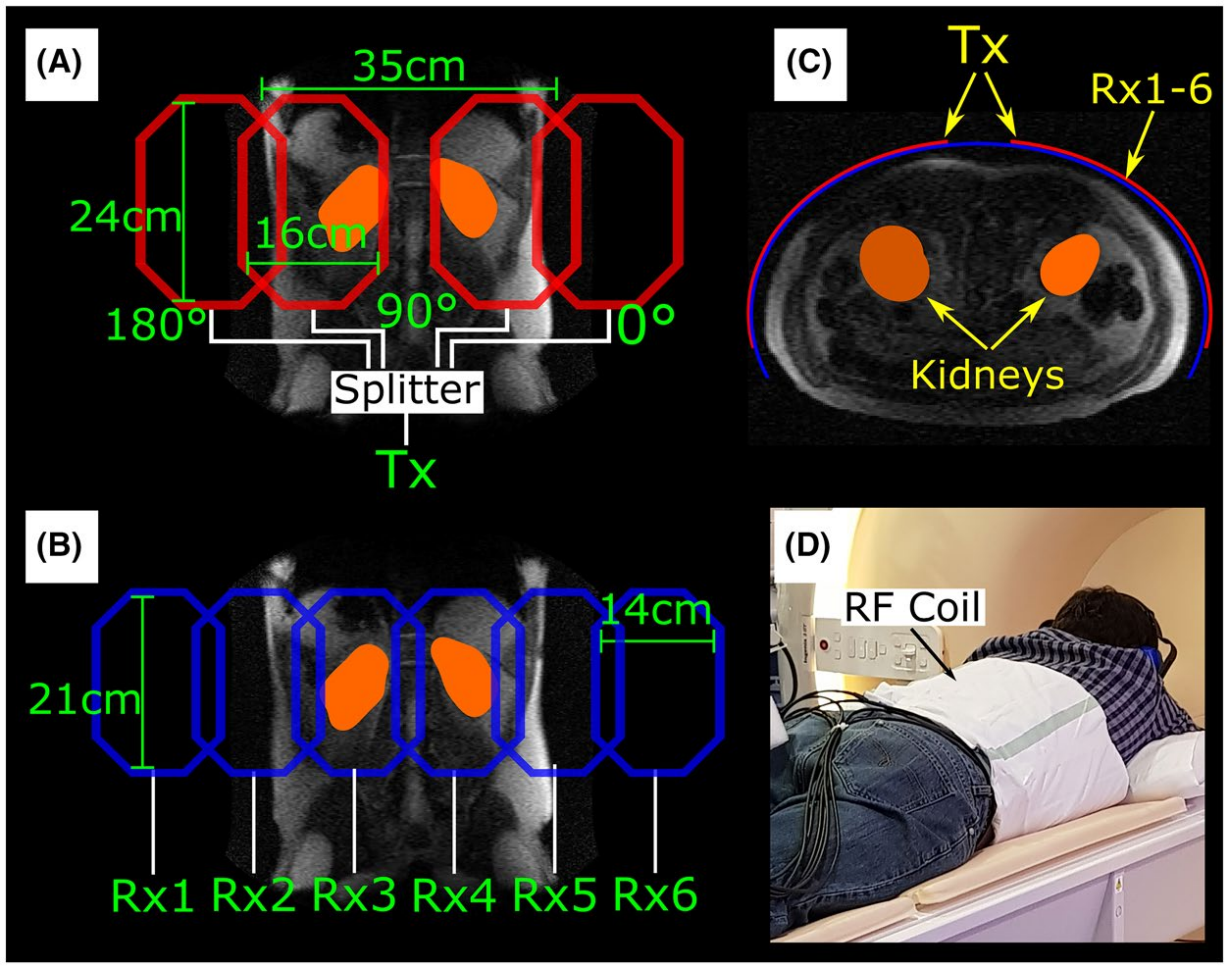
Transmit‐only receive‐only (TORO) purpose‐built coil array used to focus RF excitation and reception over the kidneys. A, Design and dimensions of the transmit coils. B, Design and dimensions of the receive coils. C, Diagram of the positioning of the TORO array with respect to the kidneys. D, Real setup of the TORO array as used for in vivo imaging. Abbreviations: Rx, receive; and Tx, transmit

A flexible dual Helmholtz quadrature transceiver thorax coil (Clinical MR Solutions, Brookfield, WI) was used for the spectroscopy and imaging as a reference. For imaging, a TORO array was also purpose‐built with 4 transmit and 6 receive coils, each with an individual receive channel. The transmit coils were designed to minimize RF excitation outside the kidneys, to reduce depolarization of the hyperpolarized ^129^Xe in the gas phase signal reservoir in the lungs and in the dissolved phase during transit in the aorta. These coils had an octagonal shape with dimensions of 24 cm and 16 cm (in z‐axis and x‐axis). Each pair of coils was positioned over 1 kidney and driven in quadrature using a purpose‐built splitter (phases 180°‐90°‐90°‐0° from left to right). Geometric decoupling was used for neighboring coils. The distance between the centers of the quadrature pairs was 35 cm (Figure 1A,C). The design of the receive coils was aimed at maximizing the sensitivity over the kidneys. For this, 6 octagonal coils elongated in the z‐axis (21 cm and 14 cm across in the z‐axis and x‐axis) with overlap decoupling were designed. The coils were placed posteriorly close to the kidneys (Figure 1B,C). Global and local specific absorption rate was calculated to be within the specific absorption rate limits for the purpose‐built coil by measuring the output power of the scanner during the 1‐dimensional CSI and imaging sequences. The 10g averaged local specific absorption rate was estimated from simulations using the electromagnetic solver HFSS (High Frequency Electromagnetic Field Simulation) (ANSYS, Canonsburg, PA).

### 2.2 Spectroscopy

Dynamic and localized spectroscopy measurements were performed. For each volunteer, the center of the transceiver coil was aligned with the approximate center of the kidneys. The chemical shift values of the spectra were referenced to the gas phase peak of the ^129^Xe signal from the base of the lungs.

Dynamic spectroscopy was obtained using the free induction decay technique with the following parameters: TR = 2000 ms, flip angle = 60°, bandwidth = 16 kHz, samples = 512, dynamics = 20, and scan time = 40 seconds.

**Figure 2:**
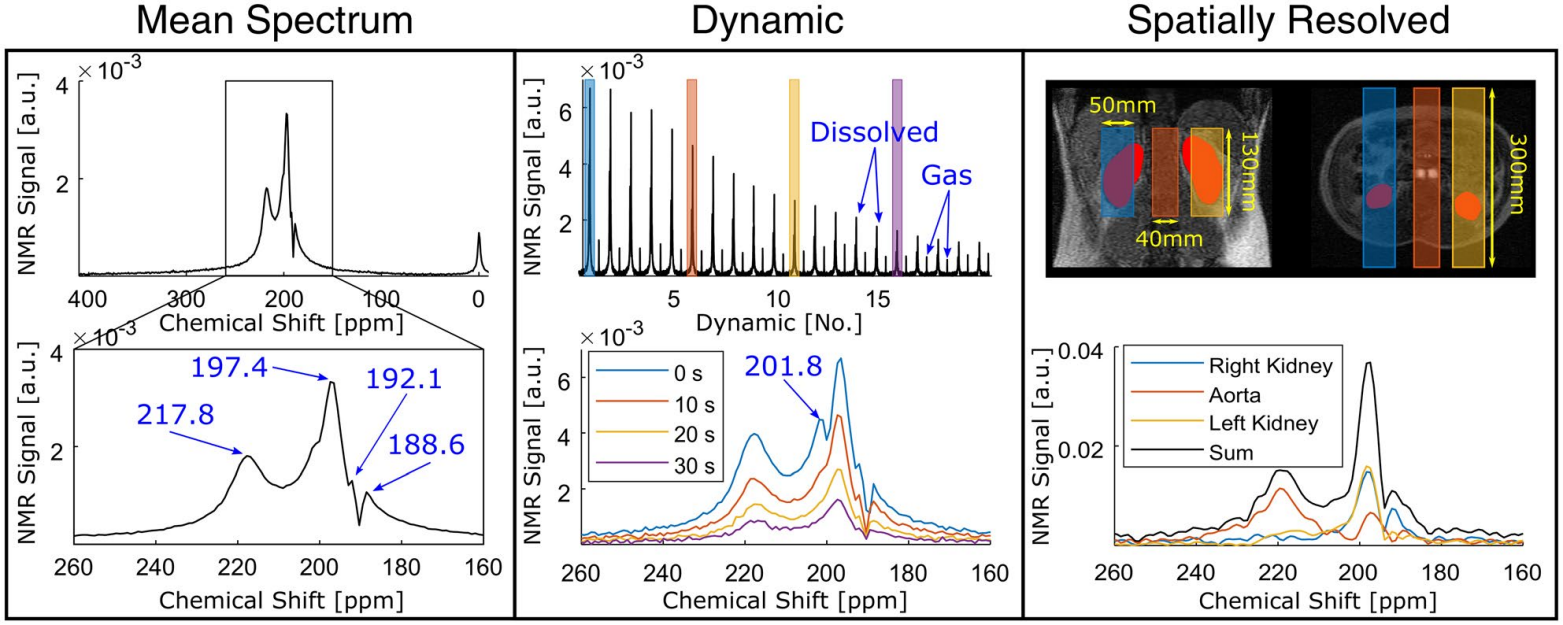
Global spectroscopy in the abdomen of a healthy volunteer and localized spectroscopy using a 1‐dimensional CSI sequence. Left: 20 global dynamics averaged. Middle: dynamic acquisitions. Right: localized spectra from selected subvolumes containing the kidneys and aorta

Spatially resolved spectroscopy was acquired using a 1‐dimensional CSI sequence within an axial slice (thickness of 130 mm) and 40 phase‐encoding steps in the left–right direction. The parameters for this acquisition were as follows: TR = 1000 ms, flip angle = 40°, bandwidth = 16 kHz, samples = 512, matrix size = 40 × 1, voxel size = 10 × 300 × 130 mm^3^, and scan time = 31 seconds. Free induction decays were low‐pass‐filtered to maintain the first 128 points. A 2D fast Fourier transform was then used to reconstruct the spectra. The spectra corresponding to the subvolume slabs containing the right kidney, aorta, and the left kidney were averaged using co‐registered proton scans for spatial reference. For the proton scans, 3 slices in each anatomical direction were scanned using the system's body coil. These reference images were acquired using a SPGR sequence with a FOV = 450 × 450 mm^2^, thickness = 15 mm, TE/TR = 4.6/6.2 ms, flip angle = 40°, and in‐plane resolution = 1.76 × 1.76 mm^2^. The evaluated subvolumes and their spatial localization with respect to the proton scans are shown in Figure 2. To assess the intersubject and intrasubject reproducibility of the spectroscopy measurements, each of the 3 volunteers underwent this measurement, and 1 of them was scanned a total of 3 times in 1 day. Here, the correlation of the maximum amplitude of the tissue peak in the left and right kidneys was calculated using the Pearson coefficient.

### 2.3 Imaging

Measurements started immediately after the inhalation of the entire xenon dose, and 17 dynamic scans were acquired per measurement every 4 seconds with an additional scan at 6 seconds using a coronal 2D SPGR sequence. The reference frequency was centered on the highest peak of the spectra in the kidneys at approximately 198 ppm from the gas peak. The following parameters were common for all imaging experiments: flip angle = 18°, bandwidth = 241.1 Hz/pixel, FOV = 560 × 560 mm^2^, thickness = 320 mm (covering the full kidney), native in‐plane resolution = 15.6 × 16.0 mm^2^, and total scan time = 1 minute.

The volunteers were scanned in the prone position using the purpose‐built TORO array (see setup in Figure 1D) and in the supine position with the flexible transceiver coil. Because of the different pulse lengths achieved for the same flip angle with the 2 coil setups, the TE and TR varied slightly: The TE/TR were 0.95/4.2 and 1.02/4.4 ms for the transceiver and TORO, respectively. Images were zero‐padded to double their apparent resolution; no additional filters were applied. The SNR of the individual channels was calculated using the background noise and for the TORO array, and the individual SNR maps were combined using a matched filter approach.^24^

A bSSFP sequence was also tested using the transceiver coil with TE and TR as similar to the SPGR sequence as possible, resulting in values for SPGR and bSSFP of TE/TR = 0.95/4.2 and 0.96/4.0 ms, respectively. Signal‐to‐noise ratio maps were calculated for each dynamic scan. Regions of interest were drawn manually in the maps over the kidneys and the lungs to track the signal evolution of the dissolved ^129^Xe.

An additional scan was obtained in 1 volunteer using a bSSFP sequence centered at approximately 218 ppm from the gas reference with a slice thickness of 200 mm. This was done to investigate differences in imaging arising from centering the frequency on different peaks of the dissolved ^129^Xe spectra.

To assess the reproducibility of the measurement, all 3 volunteers underwent imaging with the SPGR protocol, and 1 of the volunteers was scanned a total of 3 times.

## 3 RESULTS

### 3.1 Spectroscopy

**Figure 3:**
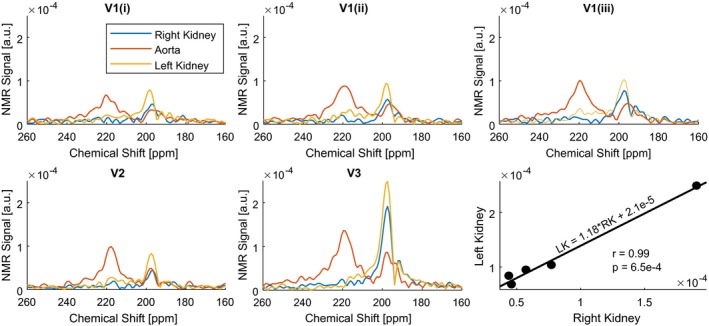
Comparison of spatially resolved spectra measured in the kidneys and the aorta of 1 volunteer (V1) measured 3 times (i, ii, iii) and the other volunteers (V2, V3). The peak amplitudes of the right kidney (RK) versus the left kidney (LK) in all scans are displayed in the bottom‐right plot, where the correlation coefficient (r) and the *p*‐value are included

The dynamic spectra when averaged over all of the acquisitions showed 4 distinct peaks at 188.6, 192.1, 197.4, and 217.8 ppm using the gas phase signal in the lungs as the 0‐ppm reference peak (Figure 2; Dynamic [Mean]). In the dynamic analysis of the global abdominal spectrum, a clear peak can be identified at 201.8 ppm in the first dynamic spectrum (Figure 2; Dynamic). In the spatially resolved spectra, the amplitude of the 197.4‐ppm peaks in both kidneys was more than 5‐fold greater in amplitude when compared with the peak at 217.8 ppm. In contrast, the 217.8‐ppm peak was a factor 1.7 times greater than the 197.4‐ppm peak from the aorta. The sum of these spatially resolved spectra resembles both the global dynamic spectrum acquired at 10 seconds and the global average spectrum (Figure 2; Spatially Resolved). In Figure 3, the repeated spatially resolved spectroscopic measurements are shown. Dominance of the 217.8‐ppm peak from the aorta and the 197.4‐ppm peak in the kidneys was observed in the spectra of all volunteers. The left kidney had approximately 18% higher ^129^Xe NMR signal than the right in all measurements. However, a significant correlation (r = 0.99, *p* = 6.5^−4^) was found between the peak amplitudes of the signals from the left and right kidneys. The highest signal amplitude was found in the 197.4‐ppm peak in the left kidney of volunteer 3, being 1.8‐fold higher than the 217.8‐ppm peak in the aorta and 2.4‐fold and 3.0‐fold higher than the maximum amplitude in the left kidney of volunteers 1 and 3, respectively.

### 3.2 Imaging

**Figure 4:**
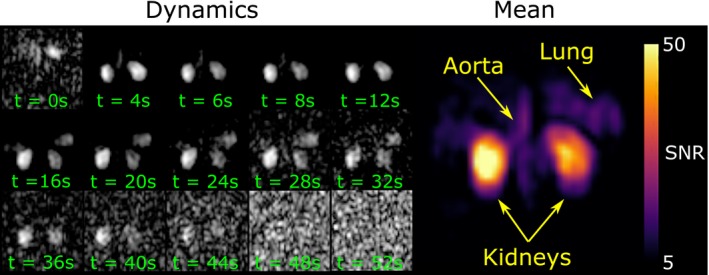
Dynamic imaging acquired with the TORO array showing hyperpolarized ^129^XE signal in the kidneys (left). The SNR of the averaged dynamics shows higher local sensitivity over the kidneys than the lungs and the aorta (right)

Using the purpose‐built TORO array, dynamic imaging was achieved in which the ^129^Xe signal in the kidneys could be observed over a time range of approximately 40 seconds. In the image formed from averaging all of the acquired dynamic images, more than a 3‐fold higher SNR was observed in the kidneys when compared with the aorta and lungs (Figure 4).

**Figure 5:**
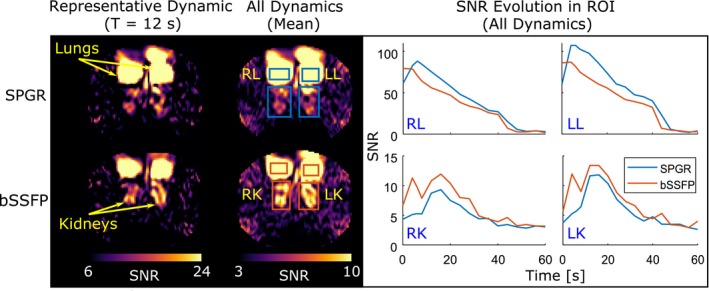
Dynamic imaging of dissolved hyperpolarized ^129^Xe in the abdomen and thorax of a volunteer using spoiled gradient echo (SPGR) and balanced SSFP (bSSFP). A representative dynamic shows the peak SNR in the kidneys. The mean shows the averaged SNR of all the dynamics. Overall, higher SNR was measured in the kidneys using bSSFP but SPGR showed higher SNR in the lungs. Similar SNR dynamics were found in each sequence for right and left lungs and kidneys. Each sequence showed different SNR evolution in the kidneys

For both tested sequences using the transceiver coil, the lungs, kidney, and aorta were clearly visible and signal dynamics were analyzed in the kidneys and in the lungs. The peak SNR in the dynamic series was found at 6 seconds in the region of interest of the left lung using SPGR (SNR = 107.3) (Figure 5, right). The SNR evolution in both lungs showed a similar decay for both sequences. In the kidneys, the overall peak SNR (13.4) yielded by the bSSFP sequence was observed at 12 seconds. Using SPGR, the peak SNR (11.8) was found in the right kidney at 16 seconds. Both kidneys showed similar uptake dynamics with the same sequence but considerably different SNR evolutions with time were observed for the 2 compared sequences (Figure 5).

**Figure 6:**
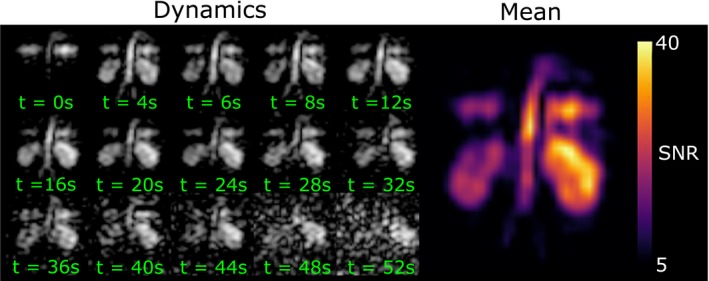
Dynamic imaging of dissolved hyperpolarized ^129^Xe in the abdomen and thorax of a volunteer using bSSFP with a center frequency aligned with the red blood cell peak found in spectroscopy

In the dynamic imaging centered approximately on the 217.8‐ppm peak in Figure 6, the signal in the lungs was visible from the first measurement taken immediately after the volunteer inhaled the volume of hyperpolarized ^129^Xe in the bag. The transit of the dissolved ^129^Xe to the kidneys is evident from the first 2 dynamic frames, and signal uptake was clearly observed in the kidneys in the second measured image acquired at 4 seconds. The ^129^Xe signal remained visible in the kidneys for more than 40 seconds.

**Figure 7:**
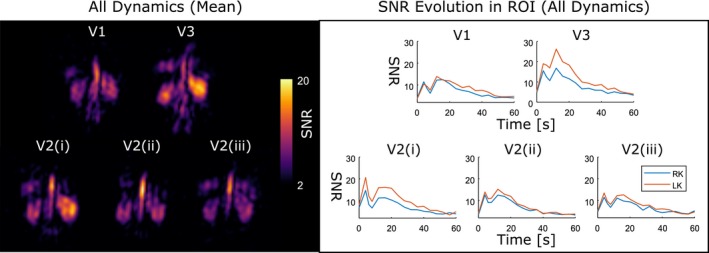
Repeated measurements using the SPGR sequence in all volunteers (V1‐V3), with volunteer 2 being scanned a total of 3 times (i, ii, and iii). The mean of all dynamics is displayed in SNR values on the left, and the mean dynamic uptake in both kidneys is shown on the right

In Figure 7, the results of the imaging reproducibility test show similar signal dynamics for all scans. This similarity is also reflected in the correlation coefficients, in which the minimum value was 0.86 between volunteer 1 and volunteer 2 (scan 2) for both kidneys. The mean of the correlation coefficient among all measurements was 0.93 for the right and 0.92 for the left kidney. The mean SNR values of the individual dynamic images varied between 6.7 (volunteer 2, scan 3) and 8.3 (volunteer 3) for the right kidney. In the left kidney, the ranges were 7.7 (volunteer 2, scan 3) to 11.8 (volunteer 3).

## 4 DISCUSSION

In this work, spectroscopy and imaging of dissolved hyperpolarized ^129^Xe in the human kidneys was presented at 3 T. From dynamic spectroscopy, an averaged signal over time showed 5 distinct dissolved phase ^129^Xe peaks from the abdomen plus the reference gas peak from the base of the lungs. To assign the peaks, we started with the 3 most accepted ones in the literature (fat/adipose tissue, plasma/soft tissue, and red blood cells) and used their relative chemical shift to identify the remaining peaks. Thus, the peak with the lowest chemical shift at 189 ppm in our spectra was assigned to fat/adipose tissue, as previously proposed.^21^, ^25^, ^26^ The peak at 197 ppm is expected to be due to xenon in interstitial fluid, correlating with the previously identified plasma/tissue.^18^, ^21^, ^25^, ^26^ In the same fashion, we assigned the peak with the highest chemical shift (218 ppm) to xenon dissolved in red blood cells, as widely accepted in the literature.^18^, ^21^, ^25^, ^26^, ^27^ The peak at 192 ppm has been reported in fewer previous publications, but due to its chemical shift between adipose tissue and interstitial fluid, we assigned it to muscles, as found in Refs [18, 25, 28]. The remaining peak at approximately 202 ppm was also initially observed by Miller et al (at 201 ppm) using a dedicated coil and was postulated to be kidney‐specific.^21^ Here, this peak was observed in the first dynamic spectrum but it then merged with the 197.4 peak from the 10‐second dynamic acquisition onward. To explain this phenomenon, we can consider that the blood delivering the xenon to the kidneys, at a first instance, reaches the cortex where the filtration occurs. Filtration is the first physiological process of the kidney and is performed based on the size of the particles (e.g., molecular weight). We can expect ^129^Xe to be filtered freely by passive diffusion, considering that its atomic weight (128.91 g/mol) is significantly less than, for example, gadolinium (604.71 g/mol), which is known to be freely filtered by the glomeruli.^29^ In this case, the xenon entering the nephron would be located in the tubules. Once there, further exchange of filtered particles with the tissue's interstitial fluid takes place due to reabsorption, which could explain the merging of the peaks. Thus, we postulate that the peak at 202 ppm is due to xenon in the kidney tubules.

The 1‐dimensional CSI sequence used in this work allowed the definition and analysis of spectra in the right and left kidneys as well as the aorta correlating with the underlying anatomy. In the regional subvolume containing the aorta, the peak red blood cell peak at 217.8 ppm dominated the spectrum, which is expected due to the high blood concentration and higher xenon solubility in red blood cells when compared to plasma.^30^ Conversely, the tissue peak (197.4 ppm) dominated the signal in the kidney subvolumes. The sum of the subvolumes qualitatively correlated well with the global spectrum of the body obtained at 20 seconds after inhalation. In the reproducibility measurements, the patterns were well‐replicated, showing dominance of the red blood cell peak in the aorta and the tissue peak in the kidneys in the spectra of all volunteers. The consistently higher signal in the left kidney is interesting; this could be a measurement bias due to, for example, coil sensitivity. However, the reproducibility of the effect—despite the significant difference in signal amplitudes observed between the volunteers—might suggest that regional physiology contributes.

The purpose‐built RF coil array showed a good spatial focus on the kidneys with respect to the surrounding tissues, and high SNR images were acquired from the dissolved hyperpolarized ^129^Xe in the kidneys. However, a noticeable trade‐off was the missing physiological information regarding the blood supply feeding the kidneys via the aorta, which could be used to estimate the arterial input function in a quantitative perfusion model. In contrast, the use of a larger flexible transceiver coil with higher coverage allowed the imaging of the aorta and the bases of the lungs, which showed a different signal evolution. This could be potentially useful to compute depolarization rates and signal input functions from the different compartments. Alternative designs and approaches with additional coils could provide efficient whole‐body coverage with increased SNR.^31^ A caveat of our RF coil array, which uses local RF transmission and reception, is that the B_1_ sensitivity could affect the dynamics of the observed signal, such as shown in Figure 4 where the uptake appears different in each kidney. A more comparable uptake between the kidneys was measured with the more homogeneous volumetric coil, which reflects the small variations in perfusion expected in healthy volunteers. In the ASL literature, these variations between left and right kidney have been reported to be at maximum 7%.^13^ Here, other factors such as the position of the kidneys within the imaging plane could also contribute to the observed inhomogeneities. Physiologically, the assessment of both kidneys is an important factor considering the known compensatory mechanism between kidneys to maintain overall filtration rates. An imbalance in uptake may reflect damage in 1 of the kidneys. Therefore, it is important to minimize or compensate for measurement biases.

In both tested imaging sequences, SPGR and bSSFP effectively imaged dissolved ^129^Xe in the lungs, aorta, and kidneys. The SNR of ^129^Xe dissolved in the lungs showed a factor 8 higher peak SNR when compared with the kidneys, which is expected, as the ^129^Xe in the lungs does not experience polarization decay during transit and the lungs contain a high reservoir of polarized gas ^129^Xe that is in constant diffusive exchange with the dissolved tissue and blood compartments. The rates of signal decay in the lungs were similar for both sequences and lungs. However, SPGR and bSSFP showed different uptake kinetics but with a similar behavior between right and left kidneys, which could be explained from the spoiling in the SPGR sequence; this will reduce the contribution from the residual magnetization after each acquisition. The observed difference in SNR evolution with time between SPGR and bSSFP will increase for spins that dissolve early and undergo several RF excitations in transit. Moreover, the peak at 4 seconds measured with bSSFP was also observed in SPGR acquisitions when the signal in the lung was decreased, which supports the rationale that the measured signal is weighted toward representing ^129^Xe spin density and not only magnetization history of the respective sequence used. Bloch simulations in future works will provide further insights into the specific signal behavior in bSSFP and SPGR sequences.^32^

Good reproducibility that was found between scans was observed with good correlations between the signal dynamics of all volunteers, but high variations in intersubject SNR were also measured. Here, it was observed that volunteer 3 showed higher SNR in both imaging and resolved spectroscopy measurements. Interestingly, volunteer 3 also had higher mean pulse rates during the experiments (1.18 beats/sec) when compared with the other 2 volunteers (0.98 and 1.00 beats/sec). Pulse rate could influence SNR, as a higher rate could allow more xenon to be delivered and accumulate in the kidneys in the TR interval between the depolarization caused by the successive RF pulses, especially for the dynamic images acquired 4 seconds apart. The influence of pulse rate, sequence timing, and mean transit times on uptake kinetics will be investigated in future works. The variations in the reproducibility tests depend on a number of factors including coil sensitivity, flip angle, sequence timings, T_1_, and T_2_ of ^129^Xe in the compartments of transit. Additionally, slight variations of the order of 10% could be expected in the efficiency of the polarization.

In this study, maximizing the dynamic information and extending the time duration of the available signal was prioritized over enhancing the image resolution. However, it is possible to reduce delays between dynamic scans and use averaging to increase the native in‐plane resolution, as we have recently shown in a study in which a resolution of 8.75 × 8.75 mm^2^ was achieved.^33^ In either case, partial volume effects arising from the full projection will average the medulla and cortex in several pixels and should be investigated in future work. The costs for the ^129^Xe, installation, and operation of the polarizer are operational and financial practicalities that should also be considered in future clinical application; they are discussed in Ref 16.

In future work, perfusion models using the dynamic uptake of hyperpolarized dissolved ^129^Xe in the kidneys will be developed and compared with DCE and ASL.

## 5 CONCLUSIONS

This preliminary study shows the feasibility of obtaining spectra and imaging of hyperpolarized dissolved ^129^Xe in the human kidney at 3 T, demonstrating the possibility of measuring parenchymal tissue perfusion. The results suggest the possibility to study not only perfusion but also early filtration in the kidney safely and noninvasively. Based on the results presented here, pilot clinical studies to assess kidney physiology using hyperpolarized ^129^Xe can be considered.
